# 双表现遗传调控药物治疗TET2基因阳性滤泡辅助T细胞表型结内外周T细胞淋巴瘤1例报告并文献复习

**DOI:** 10.3760/cma.j.issn.0253-2727.2023.07.013

**Published:** 2023-07

**Authors:** 筠渊 郎, 瑞 吕, 燕燕 宋, 德慧 邹, 刚 安

**Affiliations:** 1 中国医学科学院血液病医院（中国医学科学院血液学研究所），实验血液学国家重点实验室，国家血液系统疾病临床医学研究中心，细胞生态海河实验室，天津 300020 State Key Laboratory of Experimental Hematology, National Clinical Research Center for Blood Diseases, Haihe Laboratory of Cell Ecosystem, Institute of Hematology & Blood Diseases Hospital, Chinese Academy of Medical Science & Peking Union Medical College, Tianjin 300020, China; 2 晋城市人民医院血液科，晋城 048000 Hematology Department of Jincheng People's Hospital, Jincheng 048000, China

WHO造血与淋巴组织肿瘤新分类中定义了一类来源于滤泡辅助T细胞的特殊亚型，包括结内外周T细胞淋巴瘤伴滤泡辅助T细胞表型（nPTCL-TFH），血管免疫母细胞淋巴瘤（AITL）和滤泡T细胞淋巴瘤（F-TCL）。除表达CD4以外，nPTCL-TFH需至少表达两种以上滤泡辅助T细胞（follicular helper-T cell, TFH）标志物（PD1、CD10、BCL6、CXCL13、ICOS、MAF、SAP及CD200），组织学应具备成熟T细胞淋巴瘤的一般异型性特征，但需与AITL和F-TCL相鉴别[1]。nPTCL-TFH与AITL有共同的细胞起源[2]，因为它们的突变模式和基因表达谱非常相似，如TET2、DNMT3A、RHOA基因突变，提示nPTCL-TFH可能与AITL相关或是AITL的一种变异型[1]。我们报道1例TET2基因突变且伴TFH表型的结内外周T细胞淋巴瘤接受西达本胺和阿扎胞苷联合细胞毒药物化疗的病例，并进行文献复习，以阐明nPTCL-TFH的病理学、遗传学机制及组蛋白去乙酰化酶抑制剂（HDACi）联合DNA甲基转移酶抑制剂（DNMTi）双表观遗传治疗在nPTCL-TFH中的作用。

## 病例资料

患者，女，57岁，主因“颈部淋巴结肿大2个月”于2021年8月入院，伴有明显发热、盗汗等B症状。查体：全身散在粟粒样皮疹，颈部左侧触及数枚淋巴结。血常规正常。增强CT：颈部左侧深层、颈后、锁骨上区多发肿大淋巴结，大者短径约1.6 cm。PET/CT示：颈部双侧多发饱满淋巴结影，舌根部及口咽腔双侧壁软组织不规则增厚，代谢异常增高，考虑肿瘤浸润可能性大。患者入院后完整切除淋巴结2枚，术后病理（图1）：淋巴结结构大部分区域破坏，异型淋巴细胞弥漫增生，胞体中等至大，胞质少至中等量，胞核不规则，核分裂象易见；浆细胞较易见，散在及小灶性分布；血管增生，可见灶性坏死。免疫组化示，肿瘤细胞：CD3（+），CD5（+），CD2（+），CD7（+），BCL-6（+），CXCL13（+），ICOS（+），PD1（+），CD4（+），Ki-67阳性率40％～50％，CD30（少量+），CD20（−），PAX5（−），CD56（−），CD10（−），CD8（−）；CD21残留滤泡树突细胞（FDC）（+），CD23残留FDC（+）；Kappa部分浆细胞（+），Lambda部分浆细胞（+）。原位杂交：EBER（个别+）。淋巴结基因重排：TCRβ阳性，IGK弱阳性，IGH、TCRγ、IGL、TCRD均阴性。淋巴结基因突变（二代测序）：检测到TET2基因突变，突变位置exon3突变，氨基酸改变：p.T265Afs*13，突变频率：42.00％；TET2突变位置intron7，突变频率：6.30％。骨髓检查未见异常。患者诊断为非霍奇金淋巴瘤（nPTCL-TFH伴B细胞克隆，Ⅳ期B组，aaIPI 1分，TET2基因突变），予西达本胺+阿扎胞苷+CHOP方案（西达本胺20 mg口服，每周2次；阿扎胞苷120 mg/d皮下注射，第1～7天；环磷酰胺1.2 g静脉滴注，第2天；脂质体阿霉素20 mg静脉滴注第2天，40 mg静脉滴注第3天；长春地辛4 mg第2天，泼尼松100 mg/d，口服，第2～6天）化疗2个疗程。2个疗程化疗后CT评估及4个疗程化疗后PET/CT评估均为完全缓解。

## 讨论及文献复习

外周T细胞淋巴瘤伴滤泡辅助T细胞表型约占外周T细胞淋巴瘤，非特指型（PTCL-NOS）的41％[3]。此类型淋巴瘤的临床特征、细胞形态学、免疫组化、突变模式和基因表达谱与AITL类似，因此，2016版WHO造血与淋巴组织肿瘤修订版将其与AITL、F-TCL放在一起介绍，并命名为nPTCL-TFH[1]。nPTCL-TFH与AITL在临床表现上有许多相似之处，本例患者病史2个月，临床表现为多发淋巴结肿大伴B症状、皮疹，临床分期处于进展期，与AITL具有侵袭性且常伴明显全身症状的临床特点一致。该患者的特别之处为病变侵犯舌根部、口咽腔、扁桃体，累及韦氏环。累及韦氏环的最常见淋巴瘤类型为弥漫大B细胞淋巴瘤，其次是结外鼻型NK/T细胞淋巴瘤、IRF4相关大B细胞淋巴瘤，但在经典外周T细胞淋巴瘤及AITL中相对少见[4]–[6]，一项来自韩国的研究统计了328例韦氏环非霍奇金淋巴瘤，其中外周T细胞淋巴瘤仅占4.3％，预后方面，多因素方差分析显示，T细胞型、大于62岁及一线治疗失败可能是韦氏环淋巴瘤患者的预后不良因素[4]。

病理学方面，肿瘤细胞呈现高增殖、高凋亡的特点，免疫组化表达多种成熟T细胞标志物（CD2、CD3、CD5、CD7），CD4（+）而CD8（−），尽管其免疫组化CD10（−），但通过加做其他4种TFH标志物［BCL6（+）、CXCL13（+）、ICOS（+）、PD1（+）］证实其TFH来源明确。该患者病理结构缺乏AITL典型的分支状血管高内皮静脉增生，且CD21、CD23染色亦未见FDC网显著增生（图1），这是nPTCL与AITL的重要鉴别要点。nPTCL-TFH最常见的5种TFH标志为CD10、BCL-6、PD-1、CXCL13和ICOS[3]。近期研究认为，其证实TFH来源的敏感性从高到低依次是PD1（97％）、ICOS（94％）、CD10（44％）、CXCL13（44％）、BCL6（29％）；而特异性从高到低依次是CD10（100％）、BCL-6（100％）、CXCL13（100％）、ICOS（82％）、PD1（71％）。本例患者CD10阴性，张丹丹等[7]报道的8例韦氏环PTCL-TFH中，仅有1例表达CD10，因此CD10阴性是否为nPTCL-TFH区别于AITL的特征仍需要我们进一步探究。

**图1:**
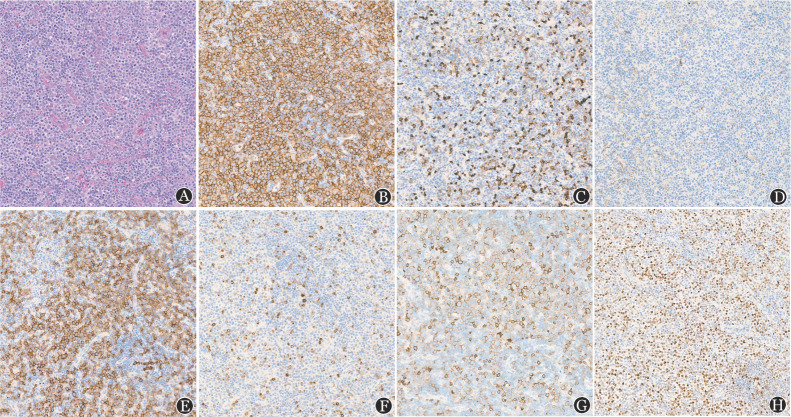
滤泡辅助T细胞表型结内外周T细胞淋巴瘤患者的病理及免疫组化染色结果 A：异型淋巴细胞弥漫增生，胞体中等至大，无明显增生的分支状高内皮静脉，滤泡树突细胞网较规则，不弥漫（HE染色）；B：CD4阳性；C：CD8阴性；D：CD10阴性；E：PD1阳性；F：CXCL13阳性；G：ICOS阳性；H：BCL6阳性

基因表达谱方面，研究证明58％～98％的nPTCL-TFH患者存在TET2基因突变，阳性率高于PTCL-NOS（2％～24％）[8]–[9]。表观遗传基因IDH2^R172^在AITL中的特征性较TET2、DNMT3A、RHOA^G17V^突变更强，而后三者也可见于其他外周T细胞淋巴瘤（PTCL）[1],[10]。本例患者仅检测到TET2突变，并未检测到IDH2^R172^，也进一步从基因学层面佐证了我们的诊断。

治疗方面，nPTCL-TFH是2016年WHO分类中最新定义的类型，尚无成熟的临床治疗方案。由于nPTCL-TFH和AITL有共同的细胞起源、相似的突变模式和基因表达谱，有学者认为治疗模式可参考AITL[11]。NCCN指南指出，CD30阴性的nPTCL-TFH一线治疗建议采用CHOP样方案。但一项meta分析显示，CHOP方案治疗PTCL患者的5年OS率仅为38.5％，其中AITL患者5年OS率为28％～36％，PTCL-NOS为45％[12]。因此，近年来，研究者们一直努力探索新的治疗方法。TET2是血液系统肿瘤的驱动因子，在造血干/祖细胞内可检测到，其通过干扰5-甲基胞嘧啶和5-羟甲基胞嘧啶的转化导致机体发生恶性淋巴瘤[13]。在PTCL-TFH中TET2基因突变频率较高，在动物实验中，TET2基因突变的小鼠可发生PTCL-TFH[14]。许多研究证明，表观遗传机制在肿瘤的发生发展中起关键作用，包括异常DNA甲基化、异常组蛋白甲基化、异常组蛋白乙酰化[15]。近期，Thienpont等[16]的研究证明肿瘤组织缺氧诱导TET基因功能丧失进而促进DNA超甲基化，因此，去甲基化治疗将是一种有希望的治疗策略。一项前瞻性研究采用DNMTi阿扎胞苷治疗携带TET2基因突变的复发难治AITL患者，ORR达到75％[17]，中位随访时间27个月，中位无进展生存时间15个月，中位总生存时间21个月。另一种表观遗传药物HDACi在PTCL-TFH中显示了独特的活性[11]，其可以诱导肿瘤细胞周期停滞、分化和细胞死亡，此外，还可以减少血管生成并调节免疫反应。在药物协同方面，研究表明，HDACi和DNA损伤剂、DNA修饰药物（阿霉素、表阿霉素、依托泊苷、顺铂、美法仑、替莫唑胺）及放疗均有协同或叠加作用，提高了DNA损伤药物的细胞毒效应[18]。多项研究采用HDACi治疗难治复发PTCL患者，其中包括AITL和nPTCL-TFH，均有较高的缓解率和较好的生存预后（表1）[11],[17],[19]–[24]，且其中一项研究还发现携带TET2、DNMT3A、RHOA基因突变的患者对治疗的反应较无上述基因突变的患者更优[11]。在上述研究基础上，2021年Falchi等[19]进行了一项多中心Ⅱ期临床研究，采用口服阿扎胞苷联合罗米司亭治疗初治外周T细胞淋巴瘤效果显著，其中具有TFH表型的PTCL显示了更高缓解率，ORR达到80％，CR率达67％。

**表1:** 组蛋白去乙酰化酶抑制剂和DNA甲基转移酶抑制剂治疗复发/难治外周T细胞淋巴瘤（PTCL）的研究

治疗方案	PTCL总例数	所有PTCL的ORR/CR率（%）	PTCL亚组（例数）	各亚组的ORR/CR率（%）	中位PFS时间（月）	中位OS时间（月）
罗米地辛[20]	130	NA	AITL（27）；PTCL-NOS（69）	30/19；29/14	29	NA
贝利司他[21]	120	25.8/10.8	AITL（22）；PTCL-NOS（77）	45.5/NA；23.3/NA	1.6	7.9
罗米地辛[22]	27	33/NA	AITL（27）	33/NA	NA	NA
西达苯胺[23]	256	39.06/10.55	AITL（137）；PTCL-NOS（192）	49.23/9.23；37.30/8.73	4.3	NA
阿扎胞苷[17]	12	75/50	AITL（12）	75/50	15	21
普拉曲沙[24]	71	52/20	AITL（20）；PTCL-NOS（34）	55/NA 50/NA	4.8	18
罗米地辛或普拉曲沙[11]	127	45.6/25	TFH-PTCL（77）	56.5/28.9	7.3	NA
阿扎胞苷和罗米地辛[19]	25	61/48	TFH-PTCL（25）	80/67	8	未达到

注 ORR：总体有效率；CR：完全缓解；NA：无数据；AITL：血管免疫母细胞淋巴瘤；PTCL-NOS：外周T细胞淋巴瘤-非特指型；TFH-PTCL：外周T细胞淋巴瘤伴滤泡辅助T细胞表型；PFS：无进展生存；OS：总生存

我们希望在HDACi（西达本胺）和DNMTi（阿扎胞苷）基础上，联合CHOP方案达到协同增效的目的。结果表明，该患者经2个疗程治疗后即达到完全缓解，而且不良反应少，耐受性佳，短期治疗效果显著，该治疗方案的远期疗效更值得我们期待。目前国内外还鲜有此方案的报道，此病例将为我们探索nPTCL-TFH新的治疗方案奠定研究基础。
